# Optimization of Newly Diagnosed Hypertension and Type II Diabetes Mellitus Before Myomectomy for Symptomatic Uterine Leiomyoma

**DOI:** 10.1002/ccr3.73327

**Published:** 2026-08-09

**Authors:** Orji Uguru Williams, Henry Eziefule Nwankwo, Ogodobiri Serena Tamarauemomoemi, Nnaji Chima Celestine, Eze Harrison Chima, Charles Caring Chihoterum

**Affiliations:** ^1^ University of Nigeria Nsukka Nigeria; ^2^ University of Chester Chester England; ^3^ Gregory University Uturu Amaidi Nigeria; ^4^ Gomel State Medical University Gomel Belarus

**Keywords:** hypertension, myomectomy, perioperative care, preoperative optimization, type 2 diabetes mellitus, uterine leiomyoma

## Abstract

Severe but newly diagnosed cardiometabolic derangements do not preclude safe myomectomy. Structured, time‐limited optimization with multidisciplinary input allows surgery to proceed once cardiovascular risk is controlled, even if glycemic targets remain imperfectly met.

## 1 Introduction

Uterine leiomyomas, also known as fibroids, are the most common benign tumors of the female reproductive tract. They arise from monoclonal proliferation of myometrial smooth muscle cells under the influence of hormonal factors, particularly estrogen and progesterone, together with genetic susceptibility and growth factor signaling pathways, and are associated with heavy menstrual bleeding, pelvic pain, anemia, infertility, and urinary symptoms [1, 2]. Treatment is individualized according to symptom severity, fibroid size and location, and fertility desires, and ranges from medical therapy with gonadotropin‐releasing hormone agonists and antagonists or selective progesterone receptor modulators to surgical and minimally invasive procedures such as myomectomy, total abdominal hysterectomy, and uterine artery embolization [2]. Fibroid prevalence and severity vary with age but are disproportionately high among young African women, underscoring the importance of safe access to uterine‐preserving surgery such as myomectomy, particularly in resource‐poor settings [1, 3].

Early identification and stabilization of newly diagnosed hypertension or diabetes before elective myomectomy is essential, since the procedure carries a substantial risk of intraoperative blood loss and postoperative complications, especially in women with large or multiple fibroids, and these comorbidities further increase perioperative risk through cardiovascular instability, impaired wound healing, and infection [4, 5, 6, 7]. Perioperative guidelines recommend careful preoperative blood pressure assessment, with many advising that elective non‐cardiac surgery be delayed when blood pressure is severely elevated, commonly using thresholds of systolic 160–180 mmHg or higher and diastolic 100–110 mmHg or higher, alongside repeated accurate measurement, evaluation for target‐organ damage, and collaborative decision‐making about whether to proceed with or delay surgery [8, 9]. Similarly, perioperative diabetes guidelines recommend early identification of diabetes, routine HbA1c testing, and individualized glycemic targets, most commonly around 100–180 mg/dL (5.6–10 mmol/L) as endorsed by the American Diabetes Association, together with withholding metformin on the day of surgery, adjusting insulin regimens to fasting status, and coordinating care across specialties to promote perioperative safety [10, 11, 12, 13, 14].

Women in sub‐Saharan Africa frequently experience delayed diagnosis of both fibroids and cardiometabolic conditions, reflecting broader gaps in routine screening and access to preventive healthcare. Integrating opportunistic screening for hypertension and diabetes into existing healthcare systems and into gynecologic preoperative pathways may therefore significantly improve surgical preparedness and long‐term outcomes [1, 3, 15]. We report the case of a woman with an extensive fibroid burden and newly diagnosed hypertension and type 2 diabetes mellitus who underwent structured preoperative optimization to enable safe myomectomy, illustrating a practical model for perioperative cardiometabolic care in a resource‐limited setting.

## 2 Case Presentation/Examination

A 40‐year‐old nulligravid woman presented with a four‐year history of infertility and progressive abdominal swelling, accompanied by occasional generalized abdominal pain but no change in her menstrual pattern; her cycles remained regular, occurring every 28 days with five days of moderate flow, and she had never previously been diagnosed with hypertension or diabetes. On examination, she had a firm, non‐tender abdominopelvic mass with a symphysio‐fundal height of 28 cm, the upper border of which lay 2 cm below the xiphisternum. Other systemic examination findings, including cardiovascular review, were essentially normal apart from an elevated blood pressure recorded at presentation.

## 3 Methods (Differential Diagnosis, Investigations and Treatment)

### 3.1 Differential Diagnosis

On the basis of the clinical findings, the differential diagnosis considered included uterine leiomyomata, an ovarian neoplasm, adenomyosis, massive pregnancy‐related uterine enlargement, and other abdominopelvic masses.

### 3.2 Investigations

At presentation, blood pressure was 170/100 mmHg with a pulse of 111 beats per minute, and random blood glucose was 306 mg/dL (17.0 mmol/L). Hemoglobin was 12.5 g/dL and platelets were 346 × 10^9^/L, with electrolytes normal except for a sodium of 147.8 mmol/L and a potassium of 5.0 mmol/L; glycated hemoglobin (HbA1c) was 6.9%. Liver function was normal, estimated glomerular filtration rate was 97 mL/min/1.73 m^2^, and both electrocardiography and urinalysis were unremarkable. Ultrasonography demonstrated a markedly enlarged uterus containing multiple intramural, subserosal, and submucosal fibroids, the largest measuring 5.73 × 4.96 cm, with normal ovaries. Taken together, these findings supported a diagnosis of symptomatic uterine leiomyomata with newly diagnosed hypertension and type 2 diabetes mellitus.

### 3.3 Preoperative Optimization and Treatment

**FIGURE 1:**
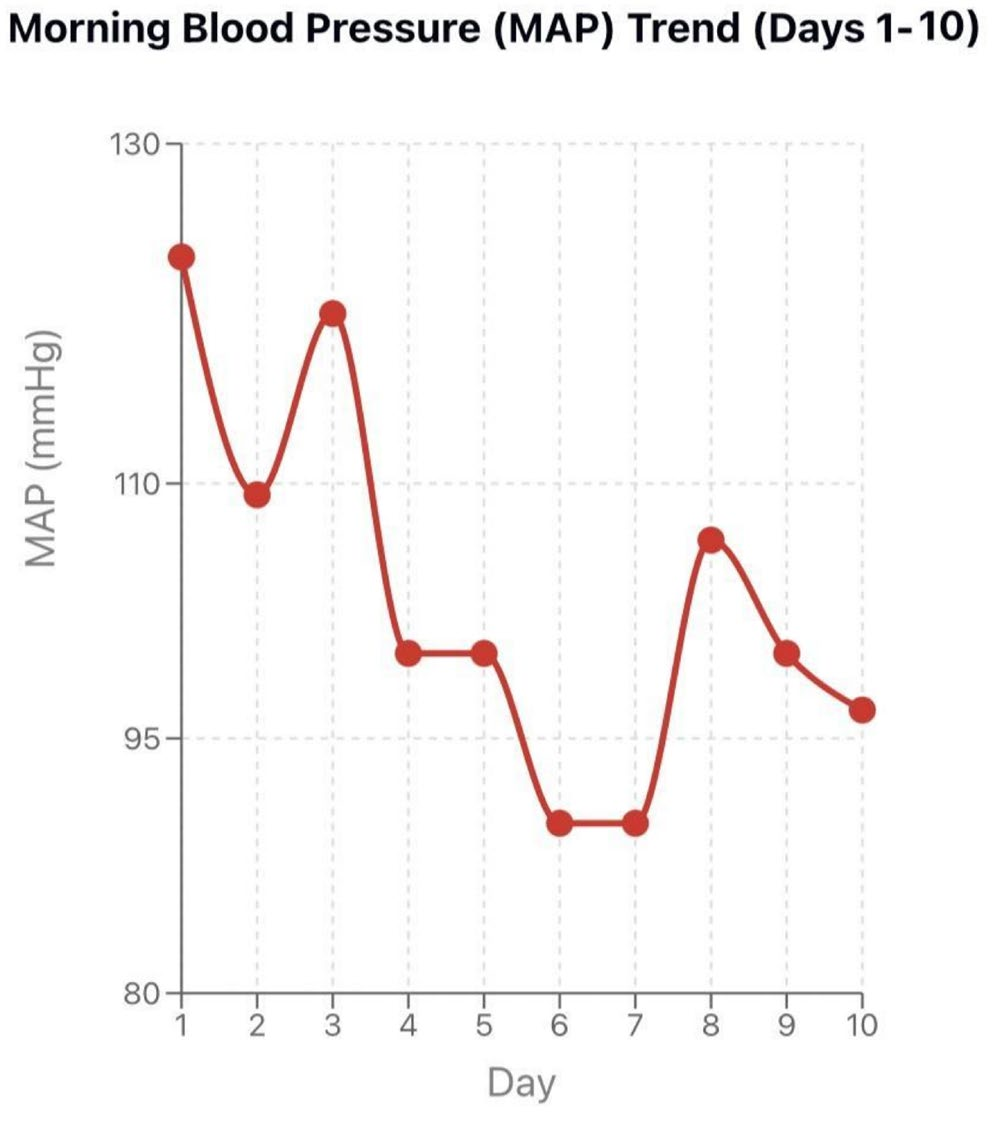
Morning blood pressure (MAP) trend. Morning mean arterial pressure fell progressively over the ten‐day optimization period, from a level consistent with stage 2 hypertension at admission to within range considered acceptable for elective surgery, with a brief plateau around Days 6–7 and intermittent elevations on Days 3 and 8 that warranted continued monitoring.

**FIGURE 2:**
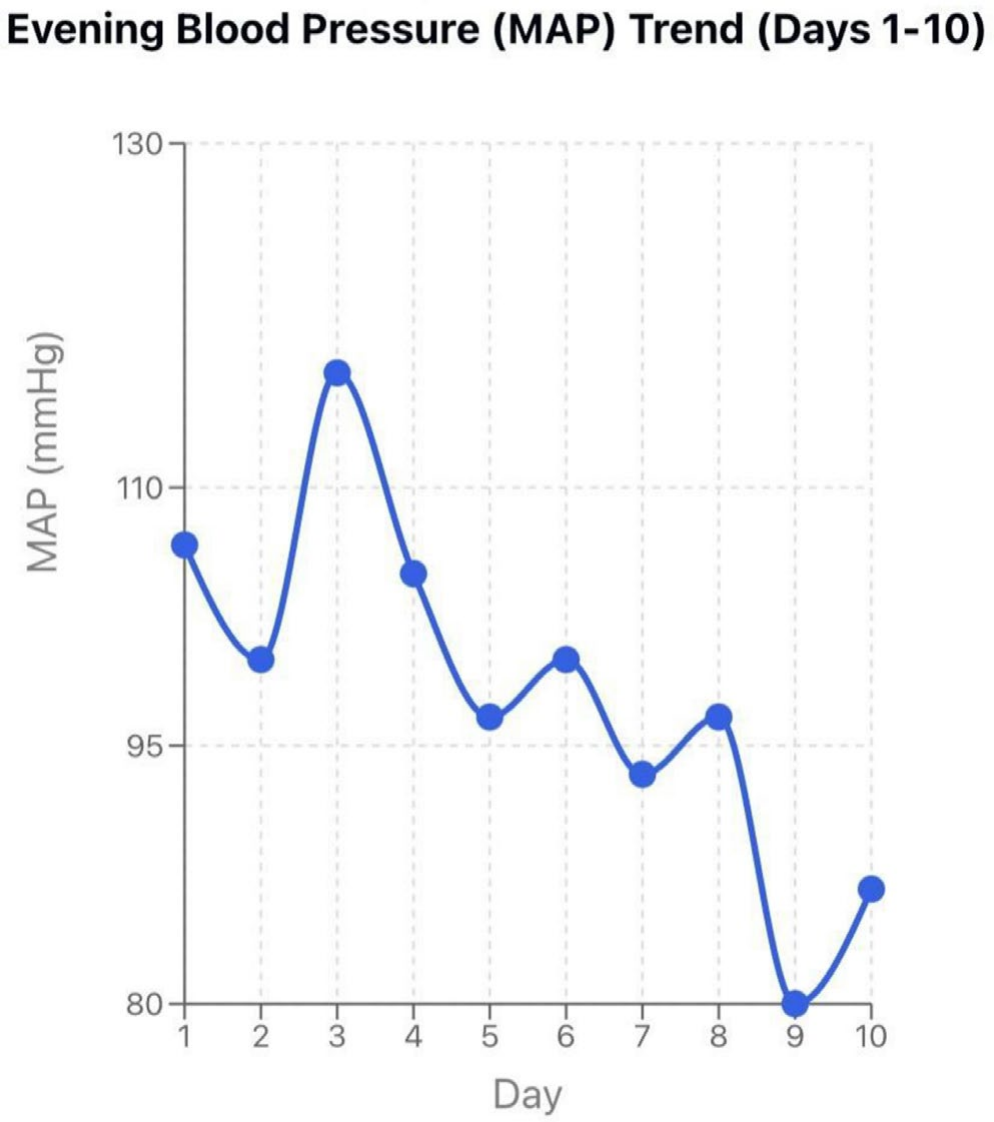
Evening blood pressure (MAP) trend. Evening MAP followed a similar downward trajectory to morning readings but was consistently lower throughout, with the most notable improvement between Days 7 and 9; this morning‐evening difference may reflect a white‐coat effect or circadian variation in blood pressure.

**FIGURE 3:**
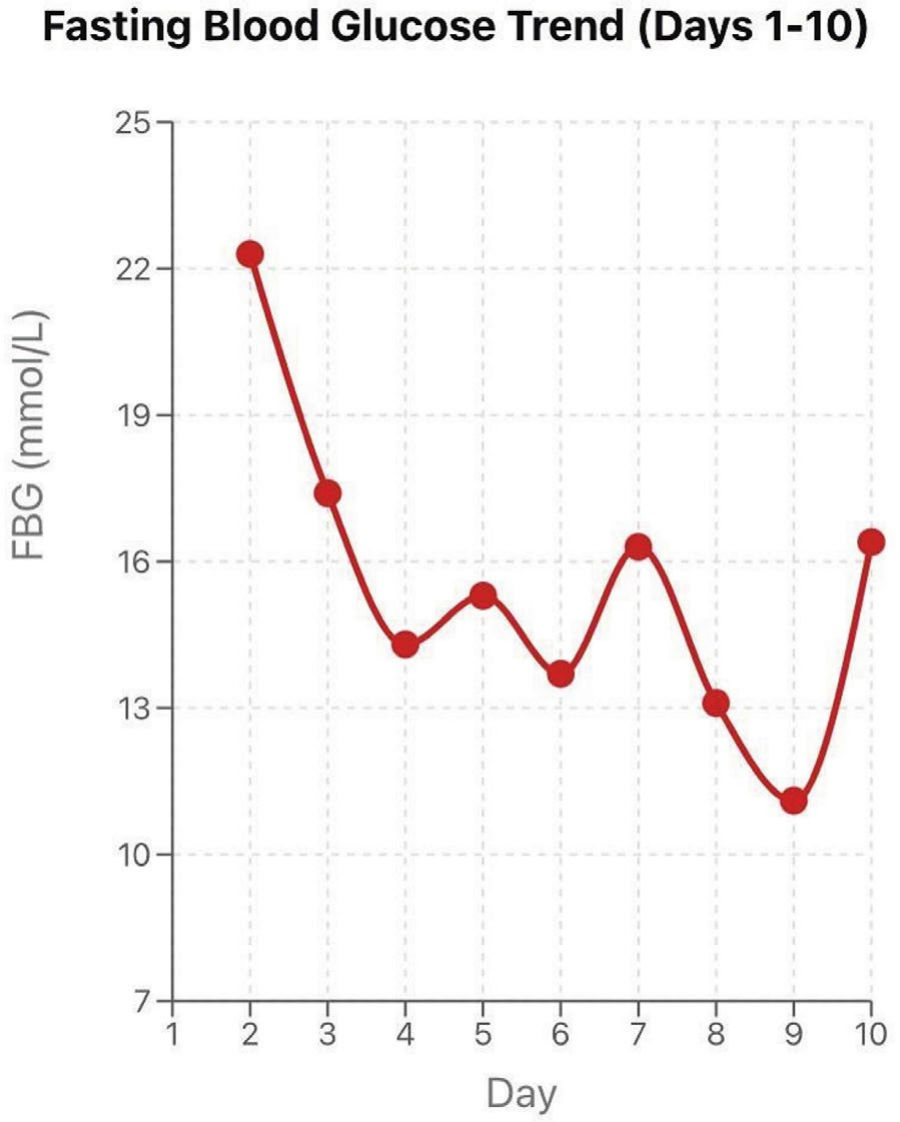
Fasting blood glucose trend. Fasting blood glucose (FBG) fell substantially from severe hyperglycemia at admission to a lower, though still above‐target, nadir by Day 9, with a minor rebound on Day 10; surgery proceeded on the endocrinologist's approval with GKI regimen the night before.

**FIGURE 4:**
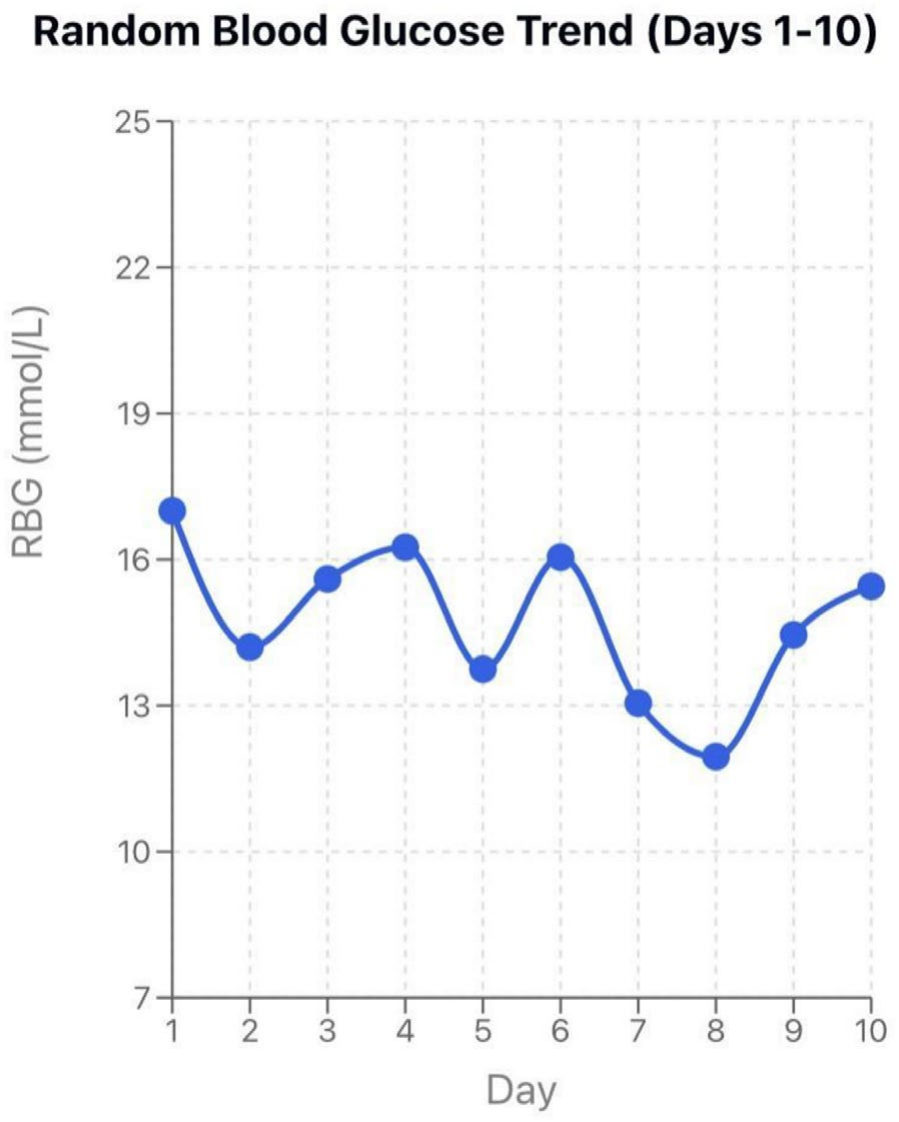
Random blood glucose trend. Random blood glucose (RBG) showed considerable day‐to‐day variability with an overall downward trend, stabilizing somewhat from Day 8 onward, but remained persistently above the target postprandial range throughout the monitoring period.

The patient was admitted for optimization and observed preoperatively for ten days. She received antihypertensive therapy with nifedipine 20 mg twice daily and hydrochlorothiazide 25 mg daily. Her initial oral antidiabetic agents, metformin and glimepiride, were discontinued following endocrinology review, and she was commenced on soluble insulin 10 IU three times daily before meals together with insulin glargine 10 IU nightly, alongside dietary counseling; glycemic targets were set at 3.3–5.6 mmol/L fasting and 5.6–8.0 mmol/L random blood glucose. Serial blood pressure and glucose monitoring over the ten‐day period showed progressive improvement, summarized in Tables 1 and 2 and illustrated in Figures 1, 2, 3, 4. 12 h before surgery, she received a glucose‐potassium‐insulin (GKI) regimen in a 10:10:10 ratio to support intraoperative glycemic stability.

**TABLE 1:** Daily fasting and random blood glucose measurements (Days 1–10).

Day	FBG (mmol/L)	RBG 1 (mmol/L)	RBG 2 (mmol/L)
1	—	—	17
2	22.3	10.2	18.2
3	17.4	13.9	17.3
4	14.3	13	19.5
5	15.3	11.8	15.7
6	13.7	15.3	16.8
7	16.3	10.2	15.9
8	13.1	10.9	13
9	11.1	14.9	14
10	16.4	17.2	13.7

**TABLE 2:** Morning and evening blood pressure measurements (Days 1–10).

Day	Morning BP (mmHg)	Evening BP (mmHg)
1	170/100	140/90
2	148/90	140/80
3	160/100	150/100
4	140/80	135/90
5	140/80	130/80
6	130/70	140/80
7	130/70	120/80
8	140/90	130/80
9	140/80	120/60
10	130/80	120/70

**FIGURE 5:**
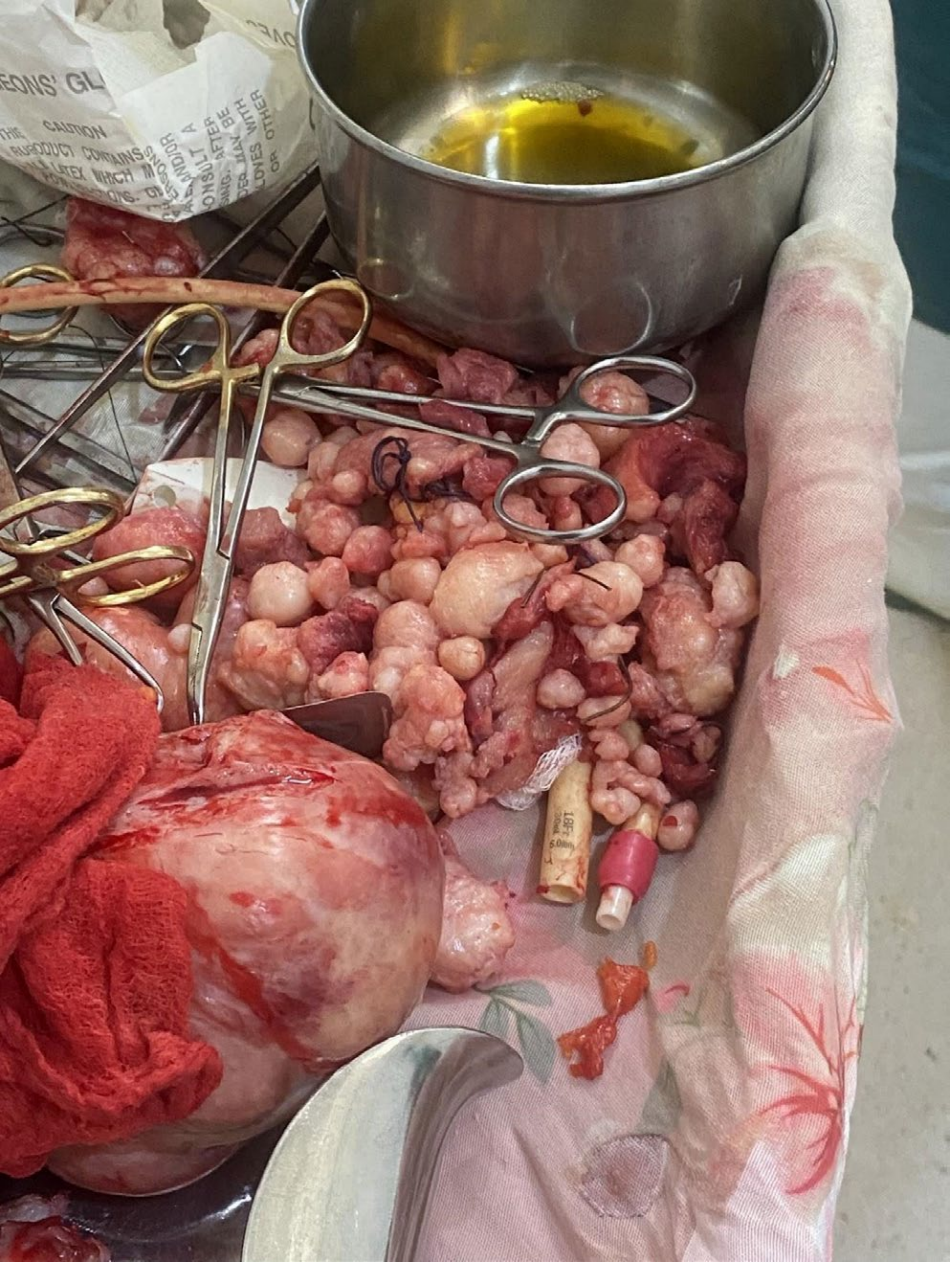
Pedunculated fibroid nodule. Intraoperative photograph showing a pedunculated fibroid nodule attached to the uterine surface by a narrow stalk near the left fallopian tube. This exophytic growth pattern is typical of subserosal leiomyomas and illustrates one of the varied morphologies encountered among the 102 fibroid nodules identified at surgery.

**FIGURE 6:**
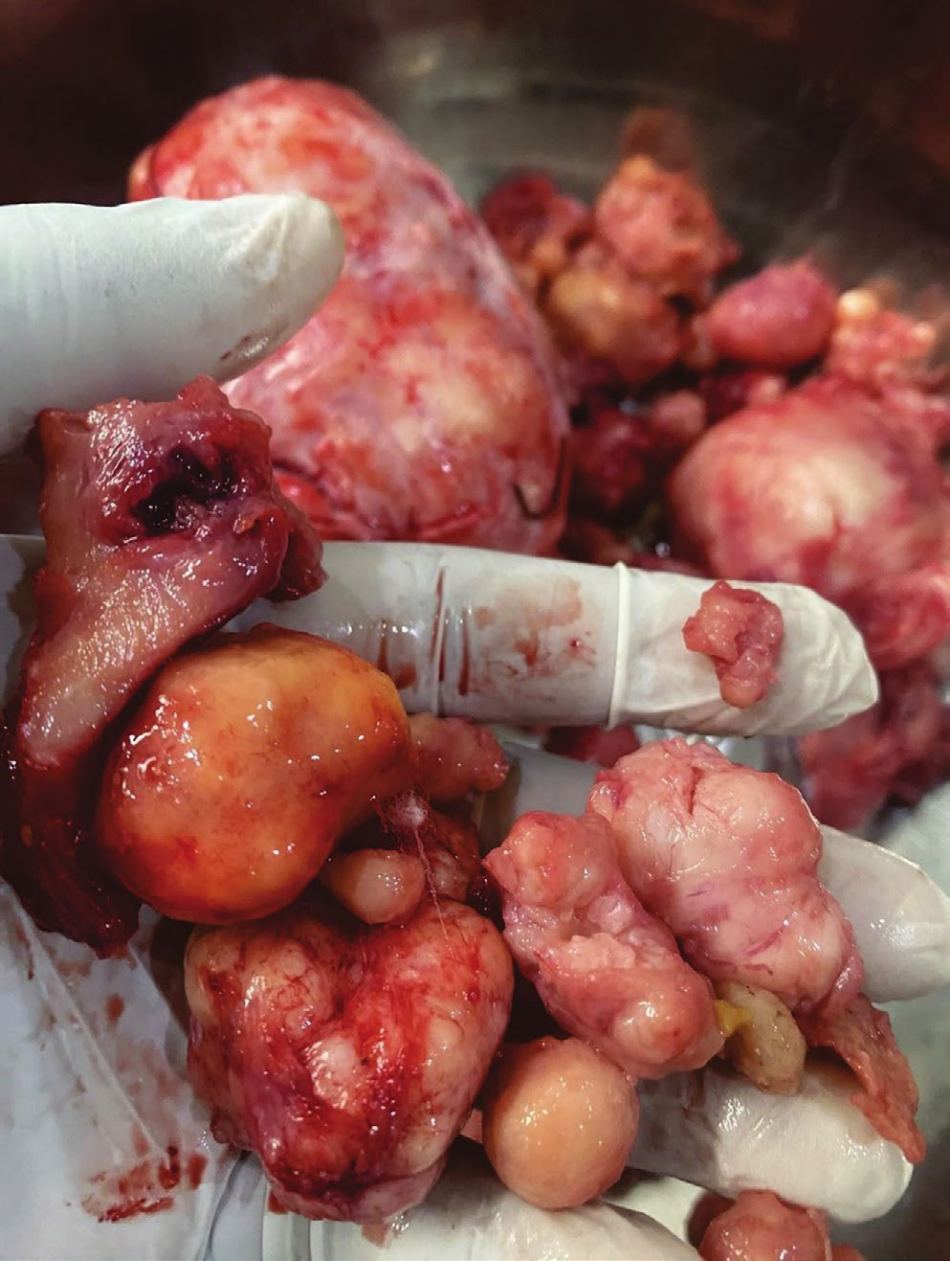
Multiple fibroid nodules. Intraoperative photograph demonstrating the extensive fibroid burden encountered at surgery, with numerous discrete nodules of varying size distorting the uterine contour. This appearance illustrates why the true fibroid count exceeded preoperative ultrasound estimates and underscores the limitations of sonography in accurately quantifying fibroid burden in markedly enlarged uteri.

**FIGURE 7:**
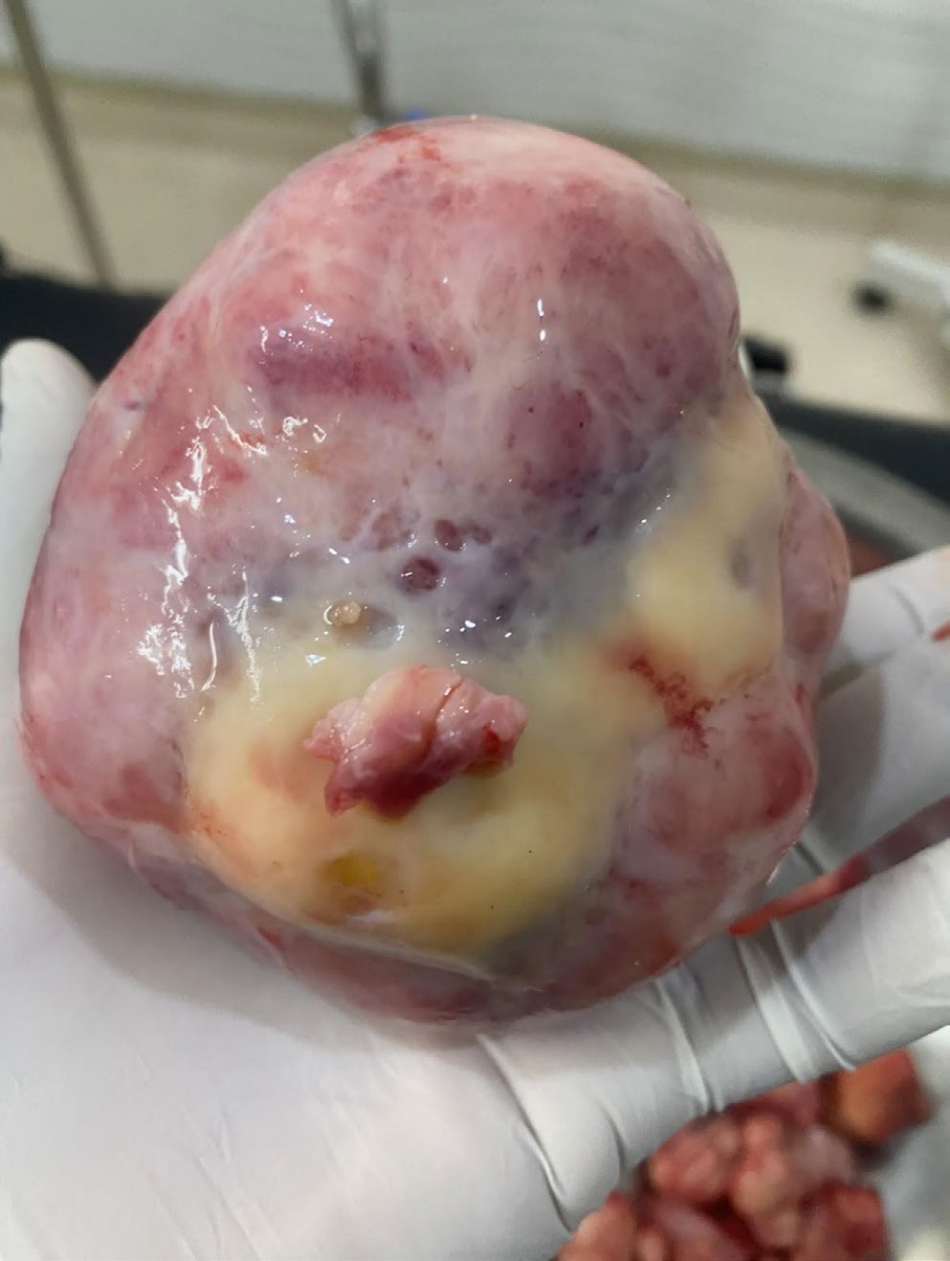
Excised myoma. Photograph of a single excised myoma showing the typical firm, well‐circumscribed, whorled cut surface characteristic of a uterine leiomyoma, which was subsequently confirmed as benign on histopathological examination with no evidence of malignancy.

## 4 Results (Outcome and Follow‐Up)

### 4.1 Surgery

She underwent open abdominal myomectomy lasting 3 h, performed via a suprapubic transverse incision under spinal anesthesia. Intraoperative findings included a massively enlarged uterus containing 102 fibroid nodules of intramural, subserosal, and submucosal types, a burden that exceeded preoperative ultrasound estimates and is consistent with the recognized limitations of sonographic assessment in significantly enlarged uteri [16]; a pedunculated fibroid near the left tube, shown in Figure 5; and numerous additional nodules of varying size, shown in Figure 6. A 22F Foley catheter was used as a tourniquet, and fibroids were removed through a hood incision. The endometrial cavity, which was breached, was repaired with Vicryl 2/0, and the serosa was closed in a baseball fashion. Estimated blood loss was 300 mL, managed with soaked mops, and the patient was transfused with one unit of whole blood. Hemostasis was secured satisfactorily.

### 4.2 Postoperative Course and Outcome

Postoperatively, she received intravenous ceftriaxone/sulbactam, gentamicin, metronidazole, and analgesics, and the GKI infusion was continued for 24 h. Postoperative packed cell volume was 30%, and the drain was removed on day five. She was discharged on postoperative day five, giving a total inpatient stay of 15 days comprising ten days of preoperative optimization and five days postoperatively. Histopathological examination of the excised fibroid nodules confirmed benign uterine leiomyomata with no evidence of malignancy, consistent with the gross appearance shown in Figure 7. At two‐week and four‐week follow‐up, the wound had healed satisfactorily and both blood pressure and random blood glucose remained stable; she was counseled regarding her fertility plans.

## 5 Discussion

This case demonstrates the feasibility of safe myomectomy in a patient with extensive uterine leiomyomata and newly diagnosed cardiometabolic comorbidities following structured preoperative optimization. Myomectomy was the appropriate surgical choice given the patient's nulligravid status and desire for fertility preservation. Open abdominal myomectomy remains an established approach for cases involving multiple or large fibroids, particularly where uterine volume and fibroid burden make laparoscopic access technically challenging, as outlined in the European Society for Gynaecological Endoscopy (ESGE) Good Practice Recommendations on fibroid surgery [16]. The intraoperative finding of 102 fibroid nodules, exceeding pre‐operative ultrasound estimates, is consistent with the well‐recognized limitations of sonographic assessment in significantly enlarged uteri, where poor acoustic windows reduce the sensitivity of transvaginal ultrasonography in detecting smaller nodules [17]. Pre‐operative counseling should therefore account for the possibility that intraoperative fibroid burden may exceed imaging estimates in such cases.

Blood pressure management in this case was successful. Over ten days of inpatient optimization with nifedipine and hydrochlorothiazide, the patient's hypertension improved from a stage 2 presentation to a final reading within the threshold considered acceptable for elective surgery under the Association of Anaesthetists guidelines [8, 18]. Preoperative evaluation for target‐organ damage is an essential component of perioperative hypertension management [19], and the absence of such damage, together with preserved renal function, supported the decision to proceed once blood pressure was adequately controlled.

Glycemic management presented the greater challenge. The severity of hyperglycemia at presentation, together with an HbA1c meeting the American Diabetes Association diagnostic threshold for diabetes, confirmed newly diagnosed type 2 diabetes and indicated a substantial glycemic burden [11]. Consistent with ADA guidance, oral antidiabetic agents were discontinued and replaced with a basal‐bolus insulin regimen, the preferred inpatient strategy under current guidelines [10, 11]. Although glucose values improved substantially over the optimization period, they remained above the recommended perioperative target throughout, a limitation we acknowledge. The decision to proceed with surgery, endorsed by the consultant endocrinologist, reflected a clinical judgment that the risks of indefinitely delaying surgery, including progressive infertility, potential anemia, and continued fibroid growth, outweighed the residual metabolic risk; intraoperative stability was further supported by a fixed‐rate GKI infusion [10]. The absence of surgical site infection and satisfactory wound healing at follow‐up support the adequacy of this approach, particularly given that both perioperative hyperglycemia and diabetes are established independent risk factors for surgical site infection [6, 7].

This case also reflects a broader challenge in sub‐Saharan Africa, where delayed presentation of both uterine fibroids and cardiometabolic conditions, driven by gaps in preventive healthcare and, in some communities, aversion to surgery, inadequate knowledge of the condition, cultural misconception, negligence, and financial constraints contribute to late presentation [20]. Integrating routine blood pressure and glucose screening into gynecological preoperative pathways, alongside targeted community health education, could identify such comorbidities earlier and improve surgical outcomes at a population level. This case provides a replicable framework for multidisciplinary perioperative care in resource‐limited settings.

## Author Contributions

**Orji Uguru Williams:** conceptualization, investigation, methodology, writing – original draft, writing – review and editing. **Henry Eziefule Nwankwo:** conceptualization, methodology, writing – original draft, writing – review and editing. **Ogodobiri Serena Tamarauemomoemi:** data curation, investigation, methodology, writing – review and editing. **Nnaji Chima Celestine:** data curation, methodology, writing – review and editing. **Eze Harrison Chima:** data curation, investigation, visualization. **Charles Caring Chihoterum:** data curation, visualization, writing – original draft.

## Funding

The authors have nothing to report.

## Consent

Written informed consent was obtained from the patient for publication of this case report and the accompanying clinical images.

## Conflicts of Interest

The authors declare no conflicts of interest.

## Data Availability

Available on request.
